# Vital Chemistry — Lectures on Animal Heat

**Published:** 1845-08-01

**Authors:** 


*Vital Chemistry — Lectures on Animal Heat, by Thomas Spencer, M. D.. Prof. Inst. and Prac. in Geneva Med. College.*—We are indebted to the author for a copy of the above, which makes a neat little volume of 144 pages, 12mo. It embraces four lectures, forming part of the author's course on the Institutes of Medicine, and their publication was by request of his class. The object is to develop the " intimate chemical relations of respiration and calorification," and contingently, those of "all the functions and phenomena of organic life." " If (as he states in his preface) every step has been fortified by ascertained facts, and the deductions have been legitimately drawn, a CIRCLE OF VITAL AFFINITIES, uniting all the structures and functions of the organism, and making each set of capillaries mutually dependent on and balanced by others, in the chemico-vital changes produced upon their passing currents of blood, has been determined." The important questions with the medical reader, are, of course, *has* every step been thus fortified, and *are* the deductions legitimately drawn? These questions we are free to confess our incompetency to answer. For those not practically familiar with analytical chemistry, the work requires more than one careful perusal to speak in any degree understandingly of its contents. The closeness with which the subject is presented, nothing being introduced but what is strictly relevant to the chain of reasoning, gives to it somewhat the character of a complicated geometrical problem, and makes it not a light undertaking to study it as it deserves. On this account those who have little taste for investigations which are prosecuted *inter taedia et labores*, will, perhaps, be repelled from its pages. Without presuming to offer an opinion as to the correctness of the positions, or the soundness of his deductions, the work bears internal evidence of the learning, industry and logical acuteness of the author. We regard the object as belonging to a department of medical inquiry, which not only possesses legitimate claims, but is of vast importance, with reference to Physiology and Pathology. If the author has been successful in his effort, he has rendered valuable service to the progress of medical truth, and will receive in due time full honor therefor; but if his argument should be shown to be defective, he will still be entitled to great credit for the acuteness which it displays, and for the enterprise ef leading the way in a province of investigation which promises rich treasures to the successful explorer.

